# Explainable Deep Learning for COVID-19 and Pneumonia Classification in Chest X-ray Images Using Layer-Wise Relevance Propagation

**DOI:** 10.7759/cureus.115532

**Published:** 2026-08-31

**Authors:** Vedant Hathalia, Tolulope Elegbede, Viktoriia Liu

**Affiliations:** 1 Computer Science and Engineering, Bellarmine College Preparatory, San Jose, USA; 2 Computer Science and Engineering, James Logan High School, Union City, USA; 3 Computer Science and Engineering, Aspiring Scholars Directed Research Program, Fremont, USA

**Keywords:** chest x-ray, convolutional neural network, covid-19, deep learning, deep taylor decomposition, explainability, explainable ai, layer-wise relevance propagation, medical imaging, pneumonia

## Abstract

Accurate and interpretable diagnosis of coronavirus disease 2019 (COVID-19) and pneumonia from chest X-ray images is critical for timely clinical decision-making, yet many deep learning models remain difficult to interpret in medical settings. In this study, a convolutional neural network (CNN) was developed to classify chest X-ray images into healthy, pneumonia, and COVID-19 categories, and its decision-making process was analyzed using explainable artificial intelligence (XAI) techniques. Layer-wise relevance propagation (LRP) and Deep Taylor Decomposition (DTD) were applied to generate attribution heatmaps identifying image regions most influential in model predictions, and multiple LRP variants were compared under both binary and multi-class classification settings. Model performance was evaluated using accuracy, precision, recall, F1-score, and confusion matrices. Explainability was assessed through complementary qualitative and quantitative analyses: heatmaps from representative clinically characterized cases were compared with reported radiographic findings, while intersection over union (IoU) quantified spatial alignment between thresholded relevance maps and manually delineated lung-field regions of interest. The binary classifier achieved 95.4% accuracy, while the three-class model achieved 95.7% overall accuracy; COVID-19 classification yielded a precision of 0.988, recall of 0.982, and F1-score of 0.985. Qualitative analysis showed that several positive-attribution methods produced relevance patterns corresponding with radiographically described pulmonary abnormalities. Quantitative IoU analysis demonstrated substantial variation in anatomical lung-field alignment across XAI methods, with the highest observed IoU values reaching approximately 0.77 at a relevance threshold of t = 0.5. Together, these results highlight the importance of combining accurate classification with interpretable model explanations for more transparent and trustworthy medical imaging applications, particularly in rural and under-resourced healthcare settings.

## Introduction

The coronavirus disease 2019 (COVID-19) pandemic, first identified in late 2019, was rapidly recognized as a global public health crisis that placed unprecedented strain on healthcare systems worldwide [1]. The rapid transmission of severe acute respiratory syndrome coronavirus 2 (SARS-CoV-2) and the emergence of multiple viral variants intensified the need for reliable diagnostic tools capable of supporting early detection and clinical decision-making [2]. Although reverse transcription polymerase chain reaction (RT-PCR) has been considered the diagnostic gold standard, limitations including test availability, turnaround time, and false negative results have motivated the exploration of complementary imaging-based approaches [3].

Chest X-ray imaging has been widely utilized in clinical practice due to its accessibility, low cost, and rapid acquisition time, making it particularly valuable in rural and under-resourced hospitals where access to advanced imaging equipment and specialized radiologists may be limited [4]. Radiographic features such as ground-glass opacities and consolidations are commonly observed in COVID-19 infection and pneumonia and can be detected on chest X-rays [5]. However, these imaging characteristics often overlap across respiratory conditions, making reliable differentiation among COVID-19, pneumonia, and healthy cases challenging, particularly during early or mild disease stages [6]. Furthermore, pneumonia encompasses multiple subtypes, including bacterial, viral, lobar, and bronchopneumonia, each of which may exhibit distinct yet sometimes subtle radiographic patterns [7]. This heterogeneity further complicates manual interpretation and increases the potential for diagnostic ambiguity [8].

Recent advances in artificial intelligence (AI) and machine learning (ML) have demonstrated strong potential for automating medical image analysis and improving diagnostic accuracy. Convolutional neural networks (CNNs), in particular, have been shown to achieve high performance in detecting pulmonary abnormalities from chest X-ray images by learning hierarchical feature representations directly from pixel data [9]. Despite these advances, CNN-based models have often been criticized for their lack of transparency, as their decision-making processes are not readily interpretable by clinicians [10]. In high-stakes medical applications, such opacity has raised concerns related to trust, bias, and clinical adoption [11].

Explainable AI (XAI) has emerged as a critical framework for addressing these concerns by providing insights into how AI models generate predictions [12]. Through XAI techniques, model behavior can be made more transparent by identifying which input features contribute most significantly to a given decision, thereby enabling validation against established clinical knowledge [13]. In medical imaging, attribution-based methods such as layer-wise relevance propagation (LRP) and Deep Taylor Decomposition (DTD) have been widely adopted to visualize regions of interest (ROIs) that influence model outputs [14]. These methods differ in the manner in which relevance is propagated through neural network layers, resulting in varying trade-offs between interpretability, stability, and sensitivity to noise [15].

Although XAI techniques have been applied in prior studies to chest X-ray classification tasks, systematic comparisons of multiple relevance propagation methods under both binary and multi-class diagnostic settings remain limited [16]. In particular, the distinction between COVID-19 and pneumonia presents a unique challenge due to overlapping radiographic features, thereby increasing the importance of interpretability analysis.

In this study, CNN-based models were evaluated for binary pneumonia classification and three-class classification of normal, pneumonia, and COVID-19 chest X-rays, and multiple LRP and DTD methods were applied to interpret model predictions. The primary objective was to evaluate classification performance across these diagnostic settings, while the secondary objective was to systematically compare the localization behavior of multiple XAI techniques. Explainability was evaluated using two complementary approaches. Qualitatively, relevance heatmaps from representative clinically characterized cases were compared with case-specific radiographic findings to assess whether model attribution corresponded with visually apparent pulmonary abnormalities. Quantitatively, intersection over union (IoU) measured spatial overlap between thresholded relevance maps and lung-field ROIs, providing a measure of anatomical alignment and off-lung attribution. Because these lung-field masks represented pulmonary anatomy rather than lesion-specific annotations, quantitative IoU was not interpreted as a direct measure of pathology-localization accuracy. Together, these analyses were designed to characterize both the predictive performance of the classifiers and the anatomical plausibility of their explanations.

## Materials and methods

Datasets

Binary Pneumonia Classification Dataset

The binary normal-versus-pneumonia classification experiment used the publicly available Chest X-ray images (pneumonia) dataset from Kaggle [17]. The version used in this study contained 5,856 image files across normal and pneumonia categories. The source dataset consists of pediatric anterior-posterior chest radiographs obtained from patients one to five years of age at Guangzhou Women and Children’s Medical Center. For the present experiment, images were partitioned at the image level using a stratified 80:20 split with a fixed random state of 42, yielding 4,684 images in the training subset and 1,172 images in the held-out subset. The held-out subset was not augmented and was used both for monitoring model performance during training and for calculation of the reported classification metrics. Because partitioning was performed at the image rather than patient level, patient-level separation between subsets could not be independently guaranteed.

Three-Class Classification Dataset

The three-class classification experiment used the publicly available COVID19+pneumonia+normal chest X-ray image dataset [18], containing 5,228 images: 1,626 COVID-19 images, 1,800 pneumonia images, and 1,802 normal images. Images were partitioned at the image level using an 80:20 stratified split. A fixed random state of 42 was used for the partitioning procedure. Images in the held-out subset were not augmented. As with the binary experiment, the image-level partitioning procedure did not enforce patient-level separation, which is acknowledged as a limitation.

Supplementary Explainability-Only Dataset

To further evaluate model attribution on clinically characterized cases, a supplementary set of 13 chest X-rays was assembled and used exclusively for post hoc explainability analysis; none of these images were included in classifier training or testing. Six images were obtained from the Covid_w/wo_pneumonia chest Xray dataset [19], comprising three COVID-19 cases with radiographic evidence of pneumonia and three COVID-19 cases without radiographic evidence of pneumonia. An additional seven chest X-ray images were obtained from six clinically characterized Radiopaedia cases [20] (rIDs 75305, 92316, 91292, 11009, 181723, and 53655). One Radiopaedia case (rID 75305) contributed two longitudinal radiographs acquired at different disease stages (Day 1 and Day 4), accounting for the seven images obtained from six case identifiers. The associated Radiopaedia case descriptions and radiographic findings were used as clinical context for qualitative comparison of model relevance patterns with reported pulmonary abnormalities.

Preprocessing and input representation

All chest X-ray images were resized to 64 x 64 pixels and loaded in red, green, and blue (RGB) format using the default settings of the Keras ImageDataGenerator, resulting in three-channel inputs with dimensions 64 x 64 x 3. Although chest X-ray images primarily convey grayscale intensity information, the original image files were stored in RGB format; therefore, no color space conversion was performed during preprocessing. Pixel values were normalized from the range 0 to 255 to the range from 0 to 1 by applying a rescaling factor of 1/255. The input resolution of 64 x 64 was selected to balance computational efficiency with preservation of broad pulmonary features relevant to classification and attribution analysis.

Data augmentation

For the binary classification model, data augmentation was applied to the training dataset using the Keras ImageDataGenerator [21]. Augmentation techniques included random shear transformations (shear range = 0.2), random zooming (zoom range = 0.2), and horizontal flipping to increase data variability and improve model generalization. All training images were additionally rescaled by a factor of 1/255. Images in the held-out subset were rescaled only, with no additional data augmentation applied.

The three-class model utilized the same augmentation strategy as the binary model, incorporating shear, zoom, and horizontal flipping, applied exclusively to the training subset. Images in the held-out subset were processed using rescaling only, without augmentation. The same augmentation parameters were applied to the training subsets of both classification experiments to maintain consistency in preprocessing.

CNN architecture

Network Design and Rationale

Both the binary and three-class models were implemented using the same convolutional backbone to maintain architectural consistency across classification tasks. The network consists of two convolutional blocks, each consisting of a 3 x 3 convolutional layer with a rectified linear unit (ReLU) activation function [22], followed by 2 x 2 max pooling layer. These layers progressively extract low-to-mid-level spatial features, including edges, textures, and localized opacity patterns associated with pulmonary abnormalities.

The resulting feature maps were flattened and passed to a fully connected dense layer with 128 neurons and ReLU activation function, enabling non-linear feature integration prior to classification. The final output layer was adapted to each classification task, with the activation function selected according to the classification objective. By maintaining a common convolutional backbone, architectural variability between the two experiments was minimized, facilitating comparison of classification and attribution behavior across tasks.

Model Specifications

Table 1 summarizes the CNN architecture used for both classification tasks. A single shared backbone was intentionally adopted to ensure architectural consistency across experiments, reducing architectural differences between experiments and facilitating comparison of performance and explainability across tasks.

**Table 1:** Convolutional neural network architecture shared across binary and three-class classification tasks. The models differ only in the final output layer to match task-specific objectives. RGB: red, green, and blue; Conv2D: two-dimensional convolutional layer; ReLU: rectified linear unit; MaxPooling2D: two-dimensional max-pooling layer

Layer	Configuration	Output shape
Input	64 × 64 RGB image	64 × 64 × 3
Conv2D	32 filters, 3 × 3, ReLU	62 × 62 × 32
MaxPooling2D	2 × 2	31 × 31 × 32
Conv2D	32 filters, 3 × 3, ReLU	29 × 29 × 32
MaxPooling2D	2 × 2	14 × 14 × 32
Flatten	-	6,272
Dense	128 units, ReLU	128
Output (2-class)	2 units, sigmoid	2
Output (3-class)	3 units, softmax	3

The network consisted of two convolutional blocks, each comprising a convolutional layer with a ReLU activation function followed by a max pooling layer. The extracted feature maps were then flattened and passed to a fully connected layer with 128 neurons. The output layer was task-specific: a two-neuron layer with sigmoid activation was used for binary classification, whereas a three-neuron layer with softmax activation was used for three-class classification. No additional architectural modifications were introduced, ensuring consistency and facilitating direct comparison across experiments.

Model training configuration

Both classification models were trained for 100 epochs using a batch size of 32 and the root mean square propagation (RMSprop) optimizer [23] with a learning rate of 0.001. For the binary classifier, binary cross-entropy was used as the loss function, and classification accuracy was monitored during training. For the three-class classifier, categorical cross-entropy was used as the loss function, with accuracy likewise monitored during training. The respective held-out subsets were provided as validation data during model fitting and were subsequently used to calculate the reported classification metrics and confusion matrices.

Explainability methods

To interpret the decision-making processes of the trained CNNs, LRP and DTD explainability methods were applied using the iNNvestigate framework [24]. These explainability techniques attribute the model’s prediction to individual input pixels, generating relevance heatmaps that highlight the regions of the chest X-ray contributing most strongly to each classification decision.

*Layer-Wise Relevance Propagation*

LRP was applied to redistribute the model’s output score by propagating relevance backward through the network layers while preserving the total amount of relevance at each step, in accordance with the relevance conservation principle. For a neuron j receiving inputs from neuron i, relevance was propagated according to the general rule:

$R_i = \sum_j \frac{z_{ij}}{\sum_{i'} z_{i'j}} R_j,\quad z_{i'j} = a_{i'}w_{i'j}$ (Equation 1),

where a_i_ is the activation, w_ij_ is the weight connecting neuron i to neuron j, and z_ij_ represents the contribution of neuron i to neuron j [25,26]. This rule distributes relevance from neuron j to its inputs in proportion to each input’s contribution to the total activation, enforcing an exact conservation of relevance across layers.

Several LRP variants were evaluated in this study, each modifying the relevance redistribution rule to emphasize different aspects of the model's decision-making process. These rules are described below and organized according to their propagation strategy.

LRP ε, also written as LRP Epsilon, adds a small stabilizing constant ε to the denominator of the base rule to prevent division by zero in layers with near-zero total activation [15]:

$R_i = \sum_j \frac{z_{ij}}{\sum_{i'} z_{i'j} + \epsilon} R_j$ (Equation 2),

where ε > 0 is a small positive constant. This modification slightly relaxes the strict conservation property of the base rule but improves numerical robustness. LRP ε tends to produce diffuse relevance maps, as the stabilizing term absorbs small relevance contributions and reduces noise from weakly activated neurons [15].

LRP Z Plus restricts propagation to only the positive part of each neuron’s contribution [15]:

$z_{ij}^{+} = \max(z_{ij}, 0)$ (Equation 3),

$R_i = \sum_j \frac{z_{ij}^{+}}{\sum_{i'} z_{i'j}^{+}} R_j$ (Equation 4).

By discarding negative contributions, LRP Z Plus produces strictly non-negative relevance maps that highlight only features supporting the predicted class. This rule is particularly well-suited to ReLU networks, where negative preactivations are suppressed, and tends to yield focused, positive-only attribution maps [15,25].

LRP Alpha Beta, also written as LRP αβ, separates positive and negative contributions into weighted streams [15,25]:

$R_i = \sum_j \left(\alpha \frac{z_{ij}^{+}}{\sum_{i'} z_{i'j}^{+}} - \beta \frac{z_{ij}^{-}}{\sum_{i'} z_{i'j}^{-}}\right) R_j$ (Equation 5),

where $z_{ij}^{+} = \max(z_{ij}, 0),\; z_{ij}^{-} = \min(z_{ij}, 0)$, and the constraint α - β = 1 preserves the conservation property [26]. The parameter α controls the weight assigned to excitatory (supporting) contributions, while β controls the weight assigned to inhibitory (contradictory) contributions. Three configurations were evaluated in this study. LRP Alpha 2 Beta 1, also written as LRP α2β1 (α = 2, β = 1), emphasizes features supporting the predicted class while retaining awareness of contradictory evidence [15]. LRP α1β0 (α = 1, β = 0), also written as LRP Alpha 1 Beta 0, discards negative contributions entirely, producing behavior equivalent to LRP Z Plus at the network level and yielding positive-only attribution maps. LRP α2β1 IB, also written as LRP Alpha 2 Beta 1 IB, applies the LRP αβ rule with α = 2, β = 1 throughout the network, and additionally applies the input-layer bounded (IB) rule at the first convolutional layer, constraining pixel-level relevance to the valid input range 0 to 1 to improve spatial precision near the image boundary [15,25,26].

LRP-Flat propagates relevance uniformly across all neurons in the preceding layer, independently of activation magnitude or connection weight [15]:

$R_i = \sum_j \frac{1}{|N_j|} R_j$ (Equation 6),

where |Nj| is the number of neurons contributing to neuron j. This rule removes all dependence on learned parameters and serves as a reference baseline, allowing assessment of whether model attribution patterns arise from feature learning rather than network architecture alone. LRP-Flat propagates relevance independently of learned activation magnitude and therefore serves as a reference propagation strategy for comparison with activation-dependent attribution methods.

LRP W Square propagates relevance in proportion to the square of each connection weight [15]:

$R_i = \sum_j \frac{w_{ij}^{2}}{\sum_{i'} w_{i'j}^{2}} R_j$ (Equation 7).

Unlike activation-dependent rules, LRP W Square depends solely on weight magnitude, making it useful in layers with near-zero activations where activation-based rules may produce vanishing relevance. However, it is insensitive to the actual input and tends to produce relevance maps that reflect network weight structure rather than input-specific pathology, limiting its interpretation for chest X-ray classification [15].

Sequential Preset rules assign different LRP propagation rules to different layers of the network in a predefined sequence, allowing each layer to use the rule best suited to its functional role [15]. Sequential Preset A applies LRP Z Plus at the first convolutional layer to ensure positive pixel-level attribution, and LRP ε at all remaining layers to improve numerical stability throughout the network. Sequential Preset B applies LRP Z Plus at the input layer, LRP αβ (α = 2, β = 1) at intermediate convolutional layers to produce signed relevance decompositions, and LRP ε at fully connected layers. This configuration produces richer signed attribution maps that retain both supporting and contradictory feature information in intermediate representations [15].

Flat initialized variants replace the input layer Z Plus rule with LRP-Flat: Sequential Preset A Flat applies LRP-Flat at the input layer and LRP ε at all other layers, while Sequential Preset B Flat applies LRP-Flat at the input layer, LRP αβ at convolutional layers, and LRP ε at fully connected layers. The Sequential Preset B Flat Until Index (IDX) variant extends this approach by applying LRP-Flat up to a specified network depth index before transitioning to the standard Preset B layer-wise assignments, offering a parametrized trade-off between flat-initialized and activation-dependent propagation [15].

Deep Taylor Decomposition

DTD provides a theoretically grounded explanation method by decomposing the network’s output as a first-order Taylor expansion around a root point x_0_, at which the model output is zero. Relevance for input feature i is defined as

$R_i = \left.\frac{\partial f(x)}{\partial x_i}\right|_{x_0}(x_i - x_{0,i})$ (Equation 8),

and is propagated recursively through the network layers using layer-specific root point constructions that satisfy the conservation property [26]. Two variants were applied in this study. The unbounded variant (Deep Taylor) places the root point at the origin without constraining the input space, allowing relevance to be propagated across the full activation range. The bounded variant (Deep Taylor Bounded) applies the LRP-IB constraint at the first network layer, restricting pixel-level relevance attribution to the valid input range from 0 to 1 [25,26]. This boundary constraint reduces spurious activation near image edges and high-contrast anatomical structures such as ribs and mediastinal boundaries, producing more spatially constrained and anatomically interpretable relevance maps.

Qualitative evaluation of attribution maps

Qualitative explainability analysis was performed on representative clinically characterized chest X-rays to assess whether relevance patterns corresponded with radiographically apparent pulmonary findings and whether substantial attribution occurred in non-pulmonary regions. For Radiopaedia-derived images, the accompanying case-specific radiographic descriptions were used as clinical context for identifying reported findings such as focal or diffuse opacities and consolidations. Attribution maps generated by the evaluated LRP and DTD methods were then visually compared with these reported findings. Qualitative assessment focused on three features: correspondence of relevance with the described pulmonary abnormality, concentration of relevance within the lung fields, and the presence of prominent attribution involving non-lung structures, image borders, or background regions. This qualitative assessment was intended to evaluate pathology-relevant spatial correspondence in representative cases and was distinct from the quantitative lung-field IoU analysis described below.

Lung-field ROI delineation

Binary lung-field ROIs were manually delineated by the study team using the visible pulmonary boundaries on each chest X-ray. For clinically characterized Radiopaedia cases, the associated case descriptions and radiographic findings provided additional clinical context during qualitative interpretation. The masks were designed to encompass the principal anatomical lung fields and to provide a reference for determining whether model relevance remained predominantly associated with pulmonary anatomy rather than obvious off-lung regions such as image borders, background areas, shoulders, or other extracorporeal structures. These ROIs were anatomical lung-field masks and were not intended to represent pixel-level segmentations of individual pathological abnormalities.

Quantitative evaluation using lung-field IoU

To quantitatively evaluate the anatomical localization of model attribution, relevance heatmaps generated by each XAI method were converted to grayscale and scaled to the range from 0 to 1. Each heatmap was then independently min-max normalized according to

$\frac{H - H_{\min}}{H_{\max} - H_{\min} + 10^{-8}}$ (Equation 9),

where H represents the heatmap intensity values. The normalized relevance maps were binarized using fixed thresholds of t = 0.5 and t = 0.7, with pixels greater than or equal to the selected threshold assigned to the relevance region. Corresponding lung-field masks were converted to binary masks using a threshold of 0.5 and resized to the heatmap dimensions using nearest-neighbor interpolation when necessary. The resulting thresholded relevance regions were then compared with the corresponding manually delineated lung-field ROIs using IoU [27].

IoU was computed according to

$IoU = \frac{|A \cap B|}{|A \cup B|}$ (Equation 10), 

where A denotes the binary relevance mask produced by an explainability method and B represents the corresponding lung region [27]. Higher IoU values indicate stronger spatial agreement between the model’s attributed regions and anatomically relevant lung areas.

Importantly, because the reference masks encompassed the anatomical lung fields rather than pathology-specific lesions, IoU was interpreted as a measure of lung-field alignment and off-lung attribution rather than lesion-level pathology-localization accuracy. Pathology-relevant spatial correspondence was considered separately through the qualitative case analysis described above.

Two threshold levels were evaluated to examine the trade-off between sensitivity and specificity in relevance localization. Lower thresholds preserve a larger fraction of attributed regions, whereas higher thresholds emphasize only the most salient relevance scores. In this research, IoU was calculated across multiple diagnostic tasks, including binary pneumonia classification, three-class classification, and COVID-19-related classification categories (COVID-19 with pneumonia (CWP) and COVID-19 without pneumonia (CWOP)).

Performance metrics and statistical analysis

Classification performance was evaluated using accuracy, precision, recall, and F1-score, with class-specific metrics derived from the corresponding confusion matrices. Quantitative anatomical alignment of model attribution was assessed using IoU, calculated as the spatial overlap between thresholded relevance maps and the corresponding lung masks. IoU values were evaluated at relevance thresholds of t = 0.5 and t = 0.7 and summarized as mean values across images for each explainability method and diagnostic task. Comparisons of IoU values across attribution methods, thresholds, and diagnostic settings were descriptive. Numerical differences were therefore interpreted as observed differences in anatomical overlap and were not treated as evidence of statistically significant differences. IoU distributions across explainability methods and diagnostic tasks were additionally visualized using violin plots at each relevance threshold to characterize the spread and distribution of anatomical lung-field overlap.

Model development, training, preprocessing, and performance evaluation were performed using Keras version 3.15.0 (Keras team, Mountain View, CA, USA), and explainability analyses were performed using iNNvestigate version 2.0.2 (iNNvestigate developers, Berlin, Germany). Data visualization was performed using Matplotlib version 3.10.3 (Matplotlib Development Team, Austin, TX, USA). The same software environment was used for both the binary and three-class classification analyses.

## Results

Training and validation performance of the CNN models

**Figure 1:**
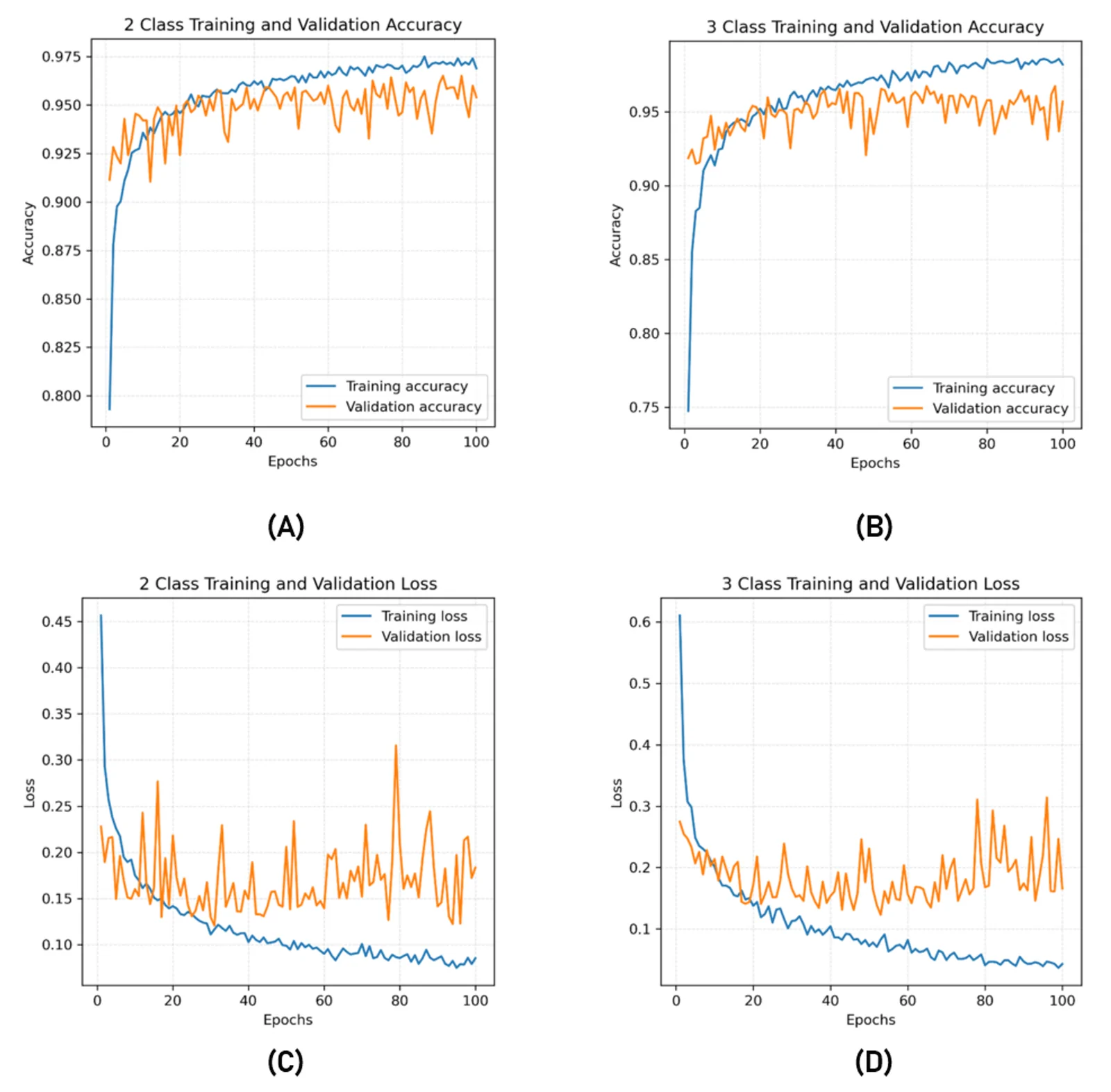
Training and validation performance of the CNN models: (A) accuracy for the two-class model, (B) accuracy for the three-class model, (C) loss for the two-class model, and (D) loss for the three-class model. CNN: convolutional neural network

Figure 1 presents the training and validation accuracy and loss curves for both the two-class and three-class CNN models. Figures 1A, 1B illustrate the accuracy curves for the two-class and three-class models, respectively. In both cases, training and validation accuracy increased steadily over the course of training and converged toward similar values, indicating successful model optimization and consistent performance between training and validation datasets. The two-class model reached stable accuracy earlier during training (Figure 1A), whereas the three-class model exhibited slightly slower convergence (Figure 1B), reflecting the increased complexity of distinguishing among three diagnostic categories.

Figures 1C, 1D present the corresponding loss curves for the two-class and three-class models, respectively. For both models, training and validation loss decreased consistently and approached a plateau during later epochs. The close agreement between training and held-out validation curves indicates similar optimization behavior across the two subsets during training, without marked divergence over the evaluated epochs.

Confusion matrix analysis

**Figure 2:**
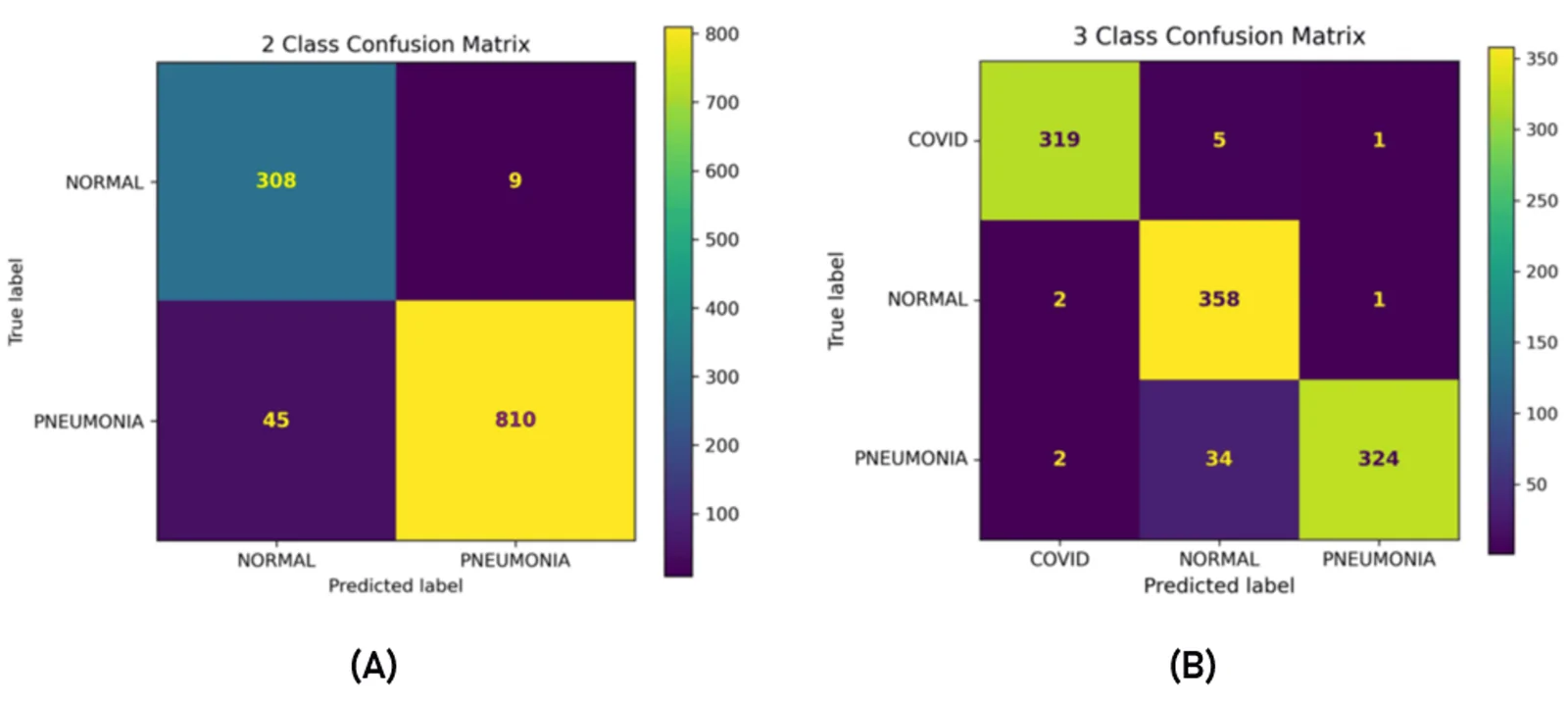
Confusion matrices for (A) the two-class classification model and (B) the three-class classification model. Rows correspond to true labels, and columns correspond to predicted labels. COVID-19: coronavirus disease 2019

Figure 2 presents the confusion matrices for the two-class and three-class classification models, providing class-level insight into prediction behavior beyond aggregate performance metrics.

In the two-class setting (Figure 2A), the majority of samples are correctly classified, with 308 healthy cases labeled as “normal” and 810 pneumonia cases correctly identified. Misclassifications are limited, with nine false positives and 45 false negatives, indicating reliable separation between pneumonia and non-pneumonia cases.

In the three-class model (Figure 2B), correct predictions dominate the diagonal entries, with 319 COVID-19, 358 normal, and 324 pneumonia images correctly classified. The largest source of error involved confusion between the normal and pneumonia classes, while misclassification involving COVID-19 was comparatively limited. Overall, the confusion matrix demonstrates strong class separation, particularly for COVID-19, with most errors occurring between the more radiographically similar normal and pneumonia categories. Although the three-class model introduces additional classification complexity, misclassifications remain largely confined to the clinically similar normal and pneumonia classes, while COVID-19 cases continue to exhibit comparatively stronger class separation.

Model performance metrics

Quantitative performance metrics for the two-class and three-class models are summarized in Table 2. The two-class model achieved an overall accuracy of 95.4%, with a pneumonia precision of 0.989, recall of 0.947, and an F1-score of 0.968, demonstrating strong performance in distinguishing pneumonia from healthy cases.

**Table 2:** Performance metrics for the two-class and three-class classification models. COVID-19: coronavirus disease 2019; F1-score: harmonic mean of precision and recall

Metric	2-class model	3-class: pneumonia	3-class: COVID-19	3-class: normal
Precision	0.989	0.994	0.988	0.902
Recall	0.947	0.900	0.982	0.992
F1-score	0.968	0.946	0.985	0.945
Accuracy	95.4%	95.7%	95.7%	95.7%

The three-class model achieved a slightly higher overall accuracy of 95.7%, while maintaining balanced performance across all diagnostic categories. COVID-19 classification yielded particularly strong results, with a precision of 0.988, recall of 0.982, and an F1-score of 0.985. Pneumonia and normal classes also demonstrated robust performance, with F1-scores of 0.946 and 0.945, respectively.

Despite the increased complexity of multi-class classification, the three-class model maintained high predictive performance while providing more detailed diagnostic differentiation. Class-wise precision, recall, and F1-scores for both models are presented in Table 2.

Qualitative analysis of explainability heatmaps

To compare qualitative explainability behavior across methods, relevance heatmaps were examined for representative chest X-rays using DTD variants and multiple LRP rules. For clinically characterized Radiopaedia cases, the accompanying radiographic descriptions were used as clinical context for comparison with visually apparent abnormalities. In the heatmaps, red regions indicate positive relevance, representing features that increase model support for the predicted class, whereas blue regions indicate negative relevance.

Qualitative assessment considered whether relevance corresponded spatially with the reported radiographic abnormality, whether attribution remained concentrated within the pulmonary fields, and whether substantial relevance appeared on non-lung structures, image borders, or background regions. This case-level analysis therefore evaluated pathology-relevant spatial correspondence and obvious off-target attribution, whereas the subsequent IoU analysis independently quantified anatomical overlap with the full lung fields.

Pneumonia Case, Two-Class Classification Model (Normal vs. Pneumonia)

**Figure 3:**
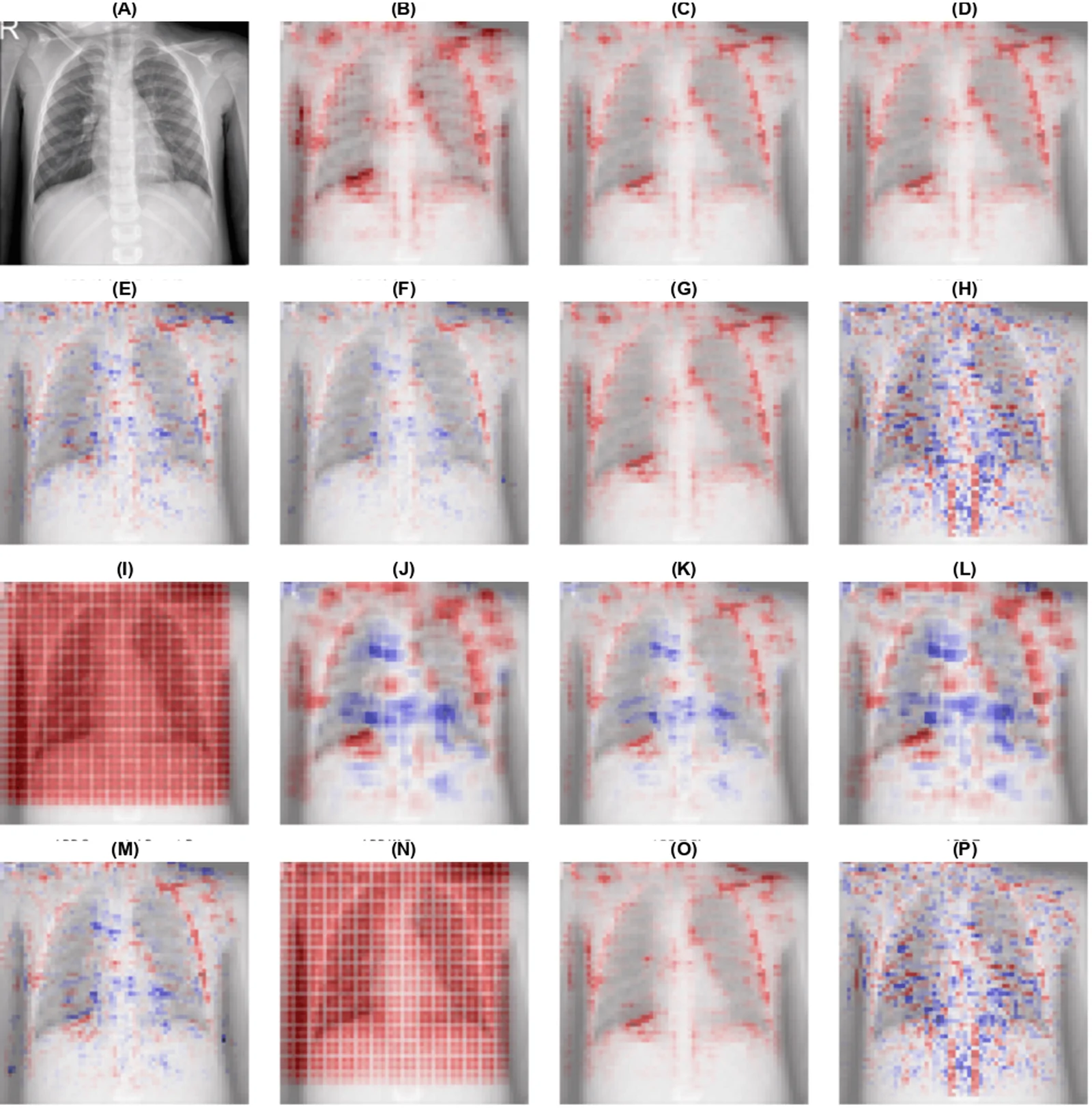
Representative pneumonia chest X-ray analyzed with Deep Taylor (bounded/unbounded) and LRP rules for the 2-class model (normal vs. pneumonia). Panels show the original image and corresponding relevance maps for each method. (A) Original chest X-ray; (B) Deep Taylor Bounded; (C) Deep Taylor; (D) LRP Alpha 1 Beta 0; (E) LRP Alpha 2 Beta 1 IB; (F) LRP Alpha 2 Beta 1; (G) LRP Alpha Beta; (H) LRP Epsilon; (I) LRP Flat; (J) LRP Sequential Preset A Flat; (K) LRP Sequential Preset A; (L) LRP Sequential Preset B Flat; (M) LRP Sequential Preset B; (N) LRP W Square; (O) LRP Z Plus; (P) LRP Z. LRP: layer-wise relevance propagation; IB: input-layer bounded Panel (A) was adapted from a case contributed by Yaïr Glick, Radiopaedia.org, rID 92316 [20], and is used under the Creative Commons Attribution-NonCommercial-ShareAlike 3.0 Unported license (CC BY-NC-SA 3.0); all relevance maps and annotations were generated by the authors.

In this pneumonia example (Figure 3), the most visually salient abnormality is a localized area of increased opacity in the lower lung field, consistent with the model’s pneumonia prediction. Several explainability methods successfully concentrated relevance within this region, whereas others assigned substantial attribution to normal anatomical structures and background regions.

Deep Taylor Bounded produced one of the cleanest localizations, with relevance concentrated in the lower lung field where the opacity is visually apparent, while suppressing activation in the background and along the image borders. This bounded constraint reduced off-lung attribution and made the explanation easier to interpret. Deep Taylor (unbounded) highlighted a similar region but exhibited more scattered activation across high-contrast structures, particularly near the rib edges and mediastinum, indicating greater sensitivity to global contrast features.

LRP α1β0 (positive-only propagation) generated a focused relevance map that remained largely within the thoracic cavity and emphasized the same lower-lung opacity. However, it also assigned mild relevance on rib contours, suggesting that the model may partially rely on edge-based cues. In contrast, the signed LRP variants (including α2β1 and α2β1-IB) produced more mixed red/blue speckling throughout the lung fields. Although some positive relevance overlapped the suspected opacity, these maps were less specific and assigned noticeable attribution to non-pathologic regions, reducing interpretability in this relatively straightforward binary classification task.

LRP Epsilon produced a diffuse and noisy relevance distribution, spreading attribution across both the lungs and surrounding non-lung regions, thereby reducing its usefulness for localizing pneumonia-related findings. LRP Flat and LRP W Square exhibited pronounced grid-like artifacts, with attribution distributed across nearly the entire image. Although portions of these maps overlapped the lung fields, the visualizations were dominated by the propagation pattern rather than pathology localization, limiting their value for clinical interpretation. LRP Z and LRP Z Plus were generally more interpretable than Epsilon, Flat, and W Square variants. In particular, LRP Z Plus maintained a clearer positive focus on the suspected abnormal region, although some sensitivity to rib edges remained. Overall, Deep Taylor Bounded, LRP α1β0, and LRP Z Plus produced the strongest qualitative correspondence with the described pulmonary findings for this case because they consistently emphasized the lower-lung opacity while limiting widespread off-target activations.

Pneumonia Case, Three-Class Classification Model (Healthy vs. Pneumonia vs. COVID-19)

**Figure 4:**
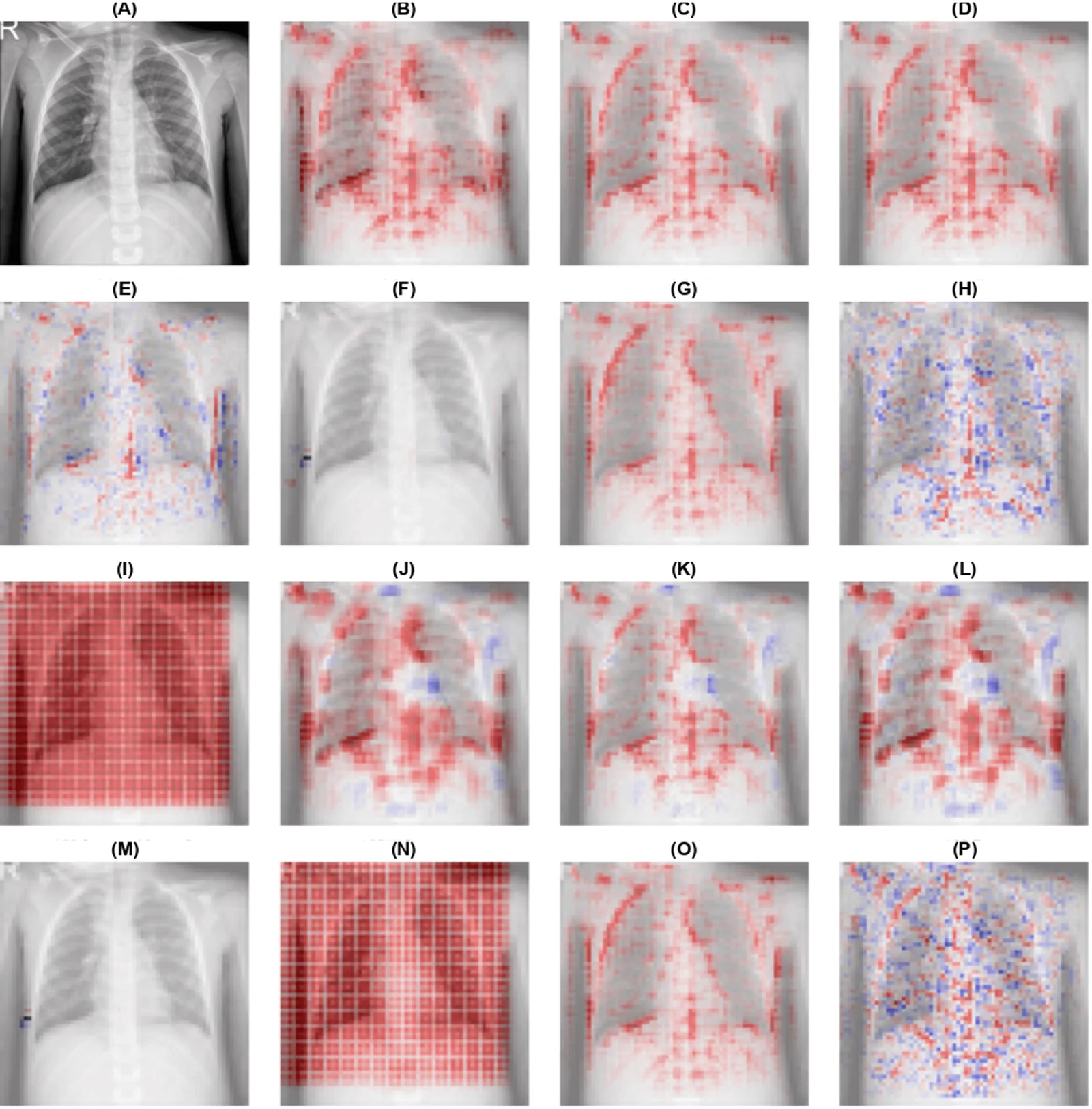
Representative pneumonia chest X-ray analyzed with Deep Taylor and LRP rules for the 3-class model (healthy, pneumonia, and COVID-19). Panels show the original image and corresponding relevance maps generated by each explanation method. (A) Original chest X-ray; (B) Deep Taylor Bounded; (C) Deep Taylor; (D) LRP Alpha 1 Beta 0; (E) LRP Alpha 2 Beta 1 IB; (F) LRP Alpha 2 Beta 1; (G) LRP Alpha Beta; (H) LRP Epsilon; (I) LRP Flat; (J) LRP Sequential Preset A Flat; (K) LRP Sequential Preset A; (L) LRP Sequential Preset B Flat; (M) LRP Sequential Preset B; (N) LRP W Square; (O) LRP Z Plus; (P) LRP Z. LRP: layer-wise relevance propagation; IB: input-layer bounded. Panel (A) was adapted from a case contributed by Yaïr Glick, Radiopaedia.org, rID 92316 [20], and is used under the Creative Commons Attribution-NonCommercial-ShareAlike 3.0 Unported license (CC BY-NC-SA 3.0); all relevance maps and annotations were generated by the authors.

For the same pneumonia image evaluated under the three-class classifier (Figure 4), relevance patterns appear more distributed compared with the two-class setting. This is expected because the model must distinguish pneumonia not only from healthy lungs but also from COVID-19, which can share overlapping radiographic features.

Deep Taylor Bounded maintained relatively strong localization within the lung fields; however, the relevance was less concentrated in a single focal region compared with the two-class case. Additional activation was observed near central thoracic structures, suggesting that the model may incorporate broader anatomical patterns when differentiating pneumonia from COVID-19. Deep Taylor (unbounded) again produced broader attribution patterns, with increased sensitivity to high-contrast anatomical structures, including rib contours and mediastinal boundaries.

LRP α1β0 continued to generate a visually interpretable positive relevance map, with more widespread lung-field attribution, consistent with increased complexity of a multi-class classification task. In contrast, LRP α2β1 produced a visually weaker relevance map in this example, suggesting that the propagated relevance may be reduced due to the rule formulation or normalization effects under this model configuration. In contrast, LRP α2β1-IB showed mixed relevance patterns but remained relatively speckled, reducing visual interpretability. LRP Epsilon continued to produce diffuse explanations, distributing relevance across a large portion of the image. LRP Flat and LRP W Square showed strong propagation artifacts, including grid-like structures, which limited their interpretability. LRP Z and LRP Z Plus preserved more lung-centered relevance compared with Epsilon, Flat, and W Square, with Z Plus generally providing clearer positive attribution, although the localization remained less focal than in the two-class setting.

Qualitatively, the three-class pneumonia explanation suggests that introducing COVID-19 as a competing class results in more distributed relevance patterns across several explanation methods. This may reflect the increased challenge of identifying class-specific imaging features when pneumonia and COVID-19 share partially overlapping radiographic characteristics.

COVID-19 Without Radiographic Evidence of Pneumonia (CWOP), Three-Class Classification Model

**Figure 5:**
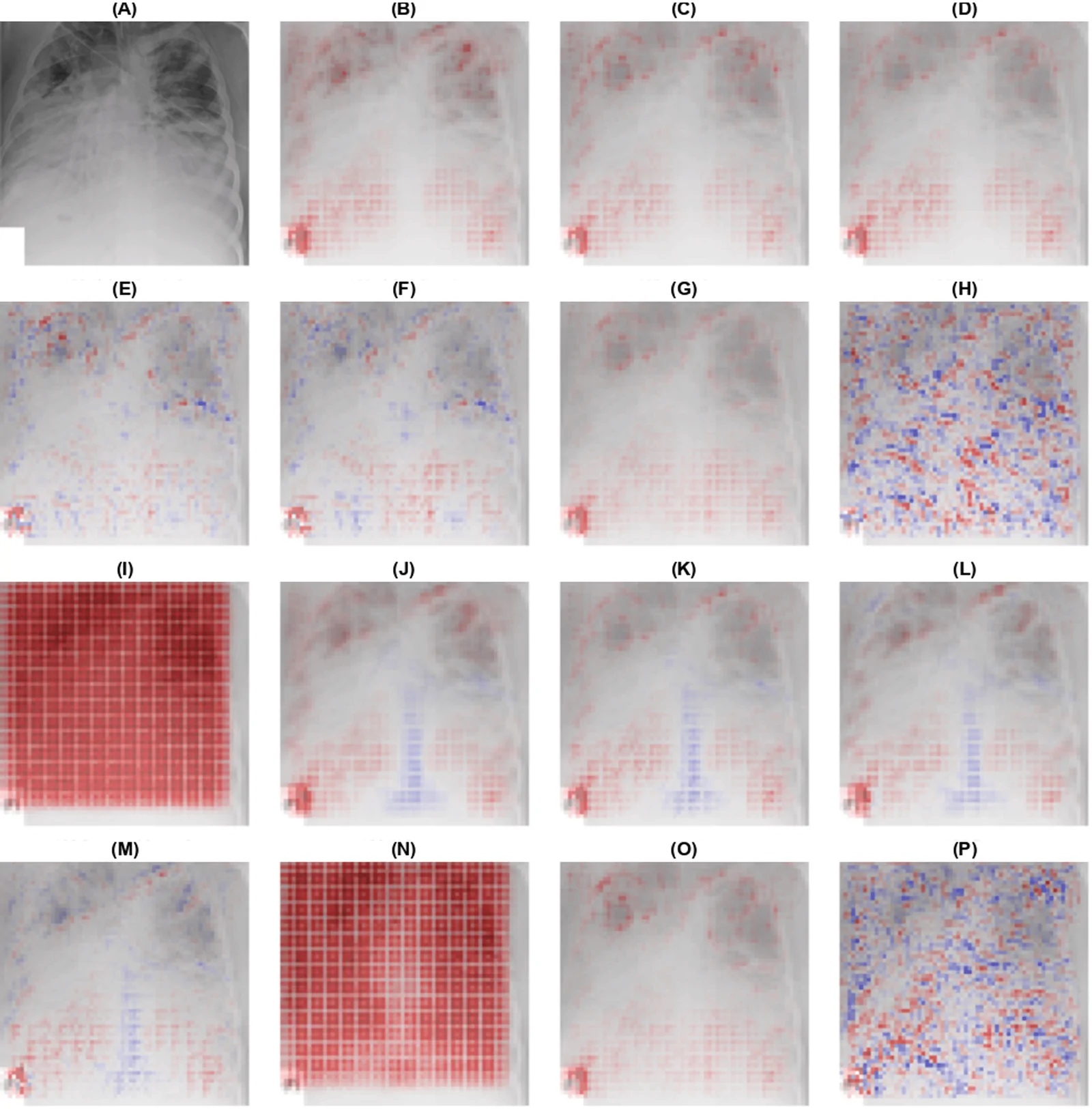
Representative CWOP chest X-ray analyzed with Deep Taylor and LRP rules for the 3-class model. Panels show the original image and corresponding relevance maps generated by each explanation method. (A) Original chest X-ray; (B) Deep Taylor Bounded; (C) Deep Taylor; (D) LRP Alpha 1 Beta 0; (E) LRP Alpha 2 Beta 1 IB; (F) LRP Alpha 2 Beta 1; (G) LRP Alpha Beta; (H) LRP Epsilon; (I) LRP Flat; (J) LRP Sequential Preset A Flat; (K) LRP Sequential Preset A; (L) LRP Sequential Preset B Flat; (M) LRP Sequential Preset B; (N) LRP W Square; (O) LRP Z Plus; (P) LRP Z. COVID-19: coronavirus disease 2019; CWOP: COVID-19 without pneumonia; LRP: layer-wise relevance propagation; IB: input-layer bounded. Panel (A) was adapted from the Covid_w/wo_pneumonia chest Xray dataset [19] and is used under the Community Data License Agreement-Sharing 1.0 (CDLA-Sharing-1.0); all relevance maps and annotations were generated by the authors.

In the CWOP example (Figure 5), the original image demonstrates diffuse bilateral increased opacity and reduced overall lung lucency, resulting in a visually denser appearance compared with the pneumonia case. For this type of presentation, effective explanations should capture distributed bilateral lung patterns while maintaining focus on relevant pulmonary regions.

Deep Taylor Bounded and Deep Taylor emphasized broad regions across both lung fields, consistent with a diffuse pattern rather than a focal pattern of image attribution. The bounded variant reduced scattered background relevance and produced a more concentrated lung-centered explanation.

LRP α1β0 also highlighted widespread relevance across the lungs, although with increased sensitivity to rib boundaries compared with Deep Taylor Bounded. Several signed-rule variants (e.g., Sequential Preset A/B) exhibited prominent structured blue contributions along the midline. These patterns suggest that negative relevance components can become visually dominant when the method separates supporting and contradicting features. While potentially informative, such explanations may be more difficult to interpret in this case because the relevance appears influenced by anatomical structures rather than predominantly by lung-field patterns. LRP Epsilon again produced highly speckled and diffuse relevance maps. LRP Flat and LRP W Square continued to show strong propagation artifacts, limiting their interpretability. LRP Z Plus generated a relatively clean positive attribution pattern across the lung regions and demonstrated better visual consistency with the diffuse radiographic appearance compared with several other signed or highly scattered rules.

Overall, the CWOP example highlights the importance of explanation methods that can represent distributed evidence rather than relying on localized regions. Deep Taylor Bounded and LRP Z Plus produced the most visually consistent lung-centered relevance patterns while minimizing prominent off-lung artifacts in this example.

COVID-19 With Radiographic Evidence of Pneumonia (CWP), Three-Class Classification Model

**Figure 6:**
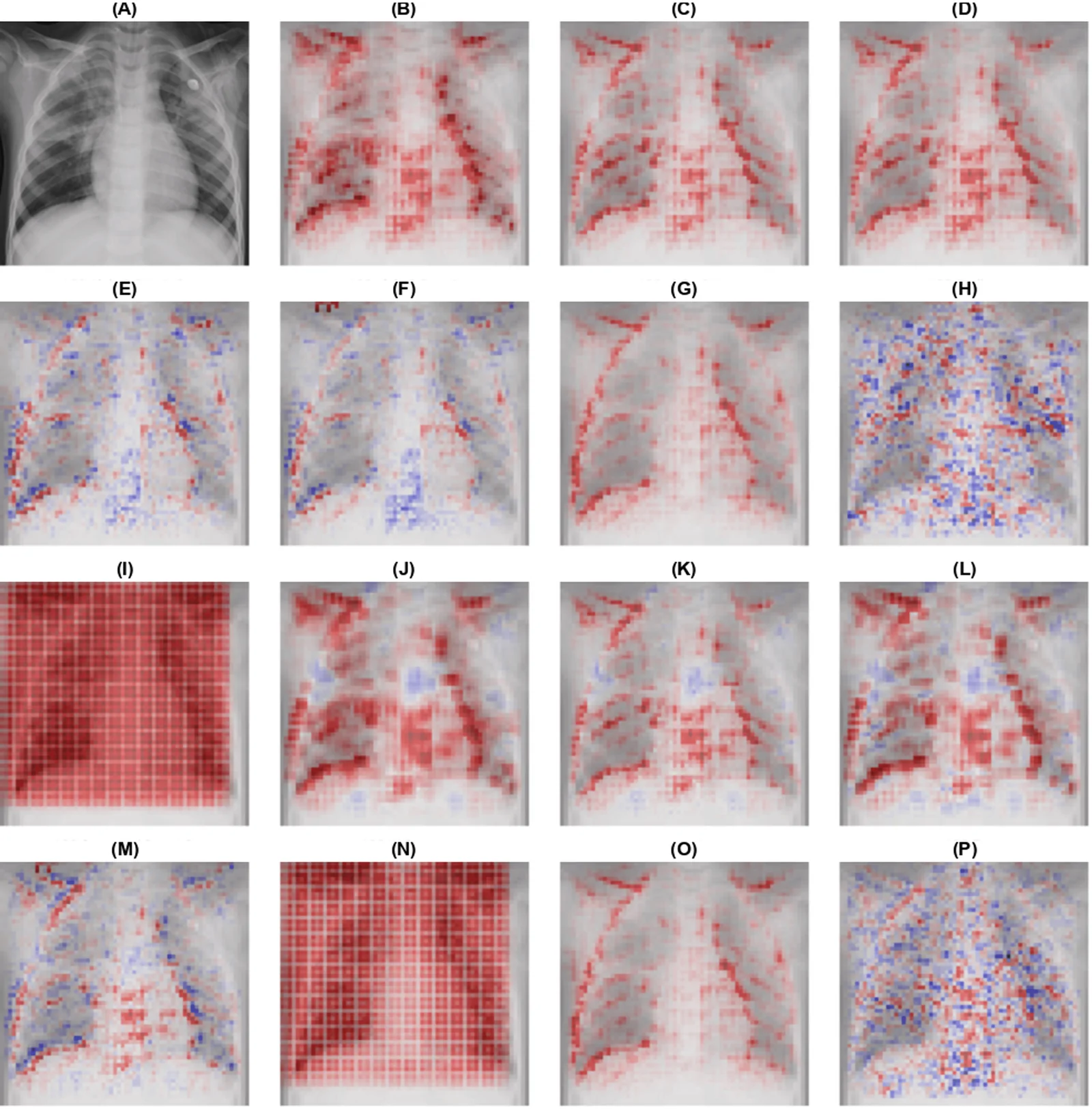
Representative CWP chest X-ray analyzed with Deep Taylor and LRP rules for the 3-class model. Panels show the original image and corresponding relevance maps for each method. (A) Original chest X-ray; (B) Deep Taylor Bounded; (C) Deep Taylor; (D) LRP Alpha 1 Beta 0; (E) LRP Alpha 2 Beta 1 IB; (F) LRP Alpha 2 Beta 1; (G) LRP Alpha Beta; (H) LRP Epsilon; (I) LRP Flat; (J) LRP Sequential Preset A Flat; (K) LRP Sequential Preset A; (L) LRP Sequential Preset B Flat; (M) LRP Sequential Preset B; (N) LRP W Square; (O) LRP Z Plus; (P) LRP Z. COVID-19: coronavirus disease 2019; CWP: COVID-19 with pneumonia; LRP: layer-wise relevance propagation; IB: input-layer bounded. Panel (A) was adapted from the Covid_w/wo_pneumonia chest Xray dataset [19] and is used under the Community Data License Agreement-Sharing 1.0 (CDLA-Sharing-1.0); all relevance maps and annotations were generated by the authors.

The CWP example (Figure 6) shows a combination of diffuse COVID-19-like changes with a more focal region of increased opacity, consistent with pneumonia overlap. Therefore, useful explanations should capture both global lung involvement and more localized high-opacity regions.

Deep Taylor Bounded provided a strong compromise: relevance highlighted broad lung regions while still emphasizing more intense attribution near the denser opacity region, and it reduced spurious activation outside the thorax. Deep Taylor and LRP α1β0 were similar but showed increased sensitivity to rib edges and central structures.

LRP αβ produced a more pronounced emphasis over the suspected higher-opacity region while also distributing relevance across the lungs, which matches the mixed-pattern nature of CWP. However, some attribution extended toward non-lung boundaries, indicating partial artifact sensitivity. LRP α2β1 and α2β1-IB again produced more mixed, speckled signed relevance; while portions overlap lung findings, the dispersion makes it harder to isolate the main pathology drivers. LRP Epsilon remained noisy; LRP Flat/W Square remained artifact-dominated. LRP Z Plus produced one of the clearer positive maps, emphasizing the lung fields and supporting the idea that positive-only emphasis can be more readable in mixed-pattern cases. Across the three-class COVID-19 cases (CWOP vs. CWP), methods that remained lung-centered while balancing focal vs. diffuse evidence-especially Deep Taylor Bounded and LRP Z Plus-were the most consistently interpretable in these representative examples.

Quantitative evaluation of explainability via lung masking and IoU analysis

**Figure 7:**
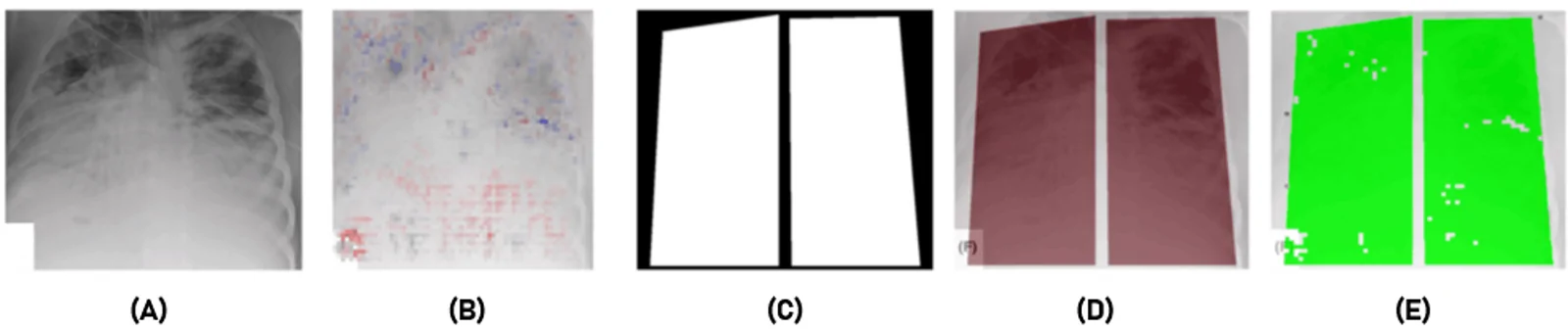
(A) COVID-19 without pneumonia chest X-ray; (B) LRP relevance map (α = 2, β = 1); (C) binary lung mask; (D) lung mask overlaid on the X-ray (region of interest); (E) IoU overlay highlighting overlap between thresholded relevance and lung regions, with the IoU value shown. COVID-19: coronavirus disease 2019; LRP: layer-wise relevance propagation; IoU: intersection over union Panel (A) was adapted from the Covid_w/wo_pneumonia chest Xray dataset [19] and is used under the Community Data License Agreement-Sharing 1.0 (CDLA-Sharing-1.0); panels (B-E) and all overlays and annotations were generated by the authors.

To complement the aggregate IoU statistics reported in Table 3, a stepwise visualization was constructed to illustrate how IoU is computed at the image level and to qualitatively validate the spatial alignment between explainability heatmaps and anatomically relevant lung regions. Figure 7 provides a representative example from the CWOP diagnostic task using the LRP α = 2, β = 1 rule.

**Table 3:** Mean IoU scores for explainability methods across diagnostic tasks and relevance thresholds (t = 0.5 and t = 0.7). IoU values quantifying spatial agreement between thresholded relevance maps and lung regions of interest are reported as means across validation images for each diagnostic task. Here, t denotes the relevance threshold used to binarize the explanation map before calculating IoU, with results reported at t = 0.5 and t = 0.7. Higher IoU indicates greater spatial overlap between thresholded model relevance and anatomically defined lung-field ROIs; it does not by itself indicate lesion-specific pathology localization. XAI: explainable artificial intelligence; LRP: layer-wise relevance propagation; IB: input-layer bounded; IDX: index; COVID-19: coronavirus disease 2019; IoU: intersection over union; t: relevance threshold used to binarize the explanation map

XAI algorithm	Pneumonia 2 classes		Pneumonia 3 classes		COVID-19 with pneumonia		COVID-19 without pneumonia	
	t = 0.5	t = 0.7	t = 0.5	t = 0.7	t = 0.5	t = 0.7	t = 0.5	t = 0.7
Deep Taylor Bounded	0.55	0.52	0.54	0.49	0.65	0.54	0.77	0.76
Deep Taylor	0.56	0.54	0.54	0.48	0.64	0.58	0.77	0.76
LRP Alpha 2 Beta 1 IB	0.57	0.54	0.56	0.55	0.67	0.64	0.77	0.76
LRP Alpha 1 Beta 0	0.56	0.54	0.53	0.47	0.64	0.57	0.77	0.76
LRP Alpha 2 Beta 1	0.57	0.57	0.57	0.57	0.68	0.65	0.77	0.76
LRP Epsilon	0.52	0.46	0.55	0.50	0.63	0.54	0.73	0.63
LRP Flat	0.11	0.07	0.11	0.07	0.14	0.09	0.13	0.07
LRP Sequential Preset A Flat	0.53	0.47	0.53	0.46	0.65	0.57	0.73	0.66
LRP Sequential Preset A	0.56	0.53	0.55	0.52	0.66	0.61	0.75	0.71
LRP Sequential Preset B Flat	0.42	0.29	0.55	0.49	0.65	0.60	0.74	0.69
LRP Sequential Preset B Flat Until IDX	0.42	0.29	0.55	0.49	0.65	0.60	0.74	0.69
LRP Sequential Preset B	0.54	0.49	0.57	0.57	0.68	0.64	0.76	0.72
LRP W Square	0.37	0.18	0.33	0.15	0.43	0.22	0.38	0.16
LRP Z Plus	0.57	0.55	0.56	0.52	0.67	0.63	0.77	0.76
LRP Z Plus Fast	0.57	0.55	0.56	0.52	0.67	0.63	0.77	0.76
LRP Z	0.52	0.46	0.54	0.47	0.64	0.54	0.72	0.63
Average	0.50	0.44	0.51	0.46	0.61	0.54	0.69	0.64

As shown in Figure 7A, the input chest X-ray exhibits diffuse bilateral lung structure without focal consolidations, consistent with the selected diagnostic category. The corresponding LRP relevance map in Figure 7B highlights regions contributing positively and negatively to the model’s prediction, with relevance distributed primarily across the bilateral lung fields and reduced activation outside thoracic anatomy.

To constrain evaluation to anatomically meaningful regions, a binary lung mask was applied (Figure 7C). This mask defines the lung ROI and serves as the reference region against which thresholded relevance maps (t = 0.7) are compared. Overlapping pixels are highlighted in green, directly visualizing the intersection between salient relevance and lung anatomy. For this example, an IoU score of 0.759 was obtained, indicating strong spatial agreement between the model’s most salient relevance attributions and anatomically defined lung regions. Higher IoU values arise when explainability methods concentrate relevance within anatomically appropriate lung regions, rather than distributing relevance diffusely across background structures or extracorporeal anatomy.

To systematically compare anatomical lung-field alignment across explainability methods and diagnostic tasks, IoU scores were aggregated in Table 3. This table reports mean IoU values computed for each explainability rule under two relevance thresholds (t = 0.5 and t = 0.7), evaluated across four diagnostic settings: two-class pneumonia classification, three-class pneumonia classification, CWP, and CWOP.

Across the evaluated diagnostic tasks, observed IoU values varied by explainability method and relevance threshold, demonstrating descriptive differences in the extent of spatial overlap between attribution maps and lung-field ROIs (Table 3). For pneumonia classification, both the two-class and three-class models exhibited moderate lung-field overlap at the lower threshold (t = 0.5), with most relevance-propagation-based methods achieving mean IoU values between 0.52 and 0.57. In contrast, methods such as LRP Flat and LRP W-Square produced substantially lower overlap (IoU ≤ 0.37), indicating diffuse or non-specific relevance attribution that extended beyond lung anatomy. Increasing the threshold to t = 0.7 generally reduced IoU across all methods, reflecting stricter retention of only the most salient relevance pixels; however, structured LRP variants such as LRP α = 2, β = 1 and LRP Z Plus maintained comparatively high overlap (IoU ≈ 0.55-0.57), suggesting greater anatomical specificity under higher selectivity.

In the evaluated images, mean lung-field overlap was higher for the COVID-19-related tasks, particularly in the CWOP category. At t = 0.5, several methods achieved mean IoU values exceeding 0.70, with LRP α = 2, β = 1, LRP Z Plus, and Deep Taylor reaching IoUs as high as 0.77. Even under the more restrictive threshold (t = 0.7), these methods retained high spatial agreement (IoU ≈ 0.70-0.76), indicating that their most salient relevance was concentrated within lung regions rather than dispersed across background structures.

In contrast, explainability methods that propagate relevance uniformly or without sign separation, such as LRP Flat, consistently produced near-zero IoU across all tasks and thresholds (IoU ≤ 0.16), underscoring their limited utility for anatomically grounded interpretation. Sequential Preset variants exhibited intermediate behavior, with Preset A outperforming Preset B, particularly in COVID-19 cases, suggesting that layer-wise propagation strategy influences the extent to which relevance remains aligned with lung anatomy.

The results in Table 3 demonstrate that positive-attribution-focused rules, particularly LRP α = 2, β = 1 and LRP Z Plus, produced among the highest observed levels of spatial alignment between model relevance and lung anatomy across both binary and multi-class diagnostic settings. The consistent decline in IoU at higher thresholds further highlights the sensitivity-specificity trade-off inherent in relevance thresholding, motivating distribution-level analysis of IoU values across methods and tasks, which is explored in the following section.

Distributional analysis of IoU across thresholds

**Figure 8:**
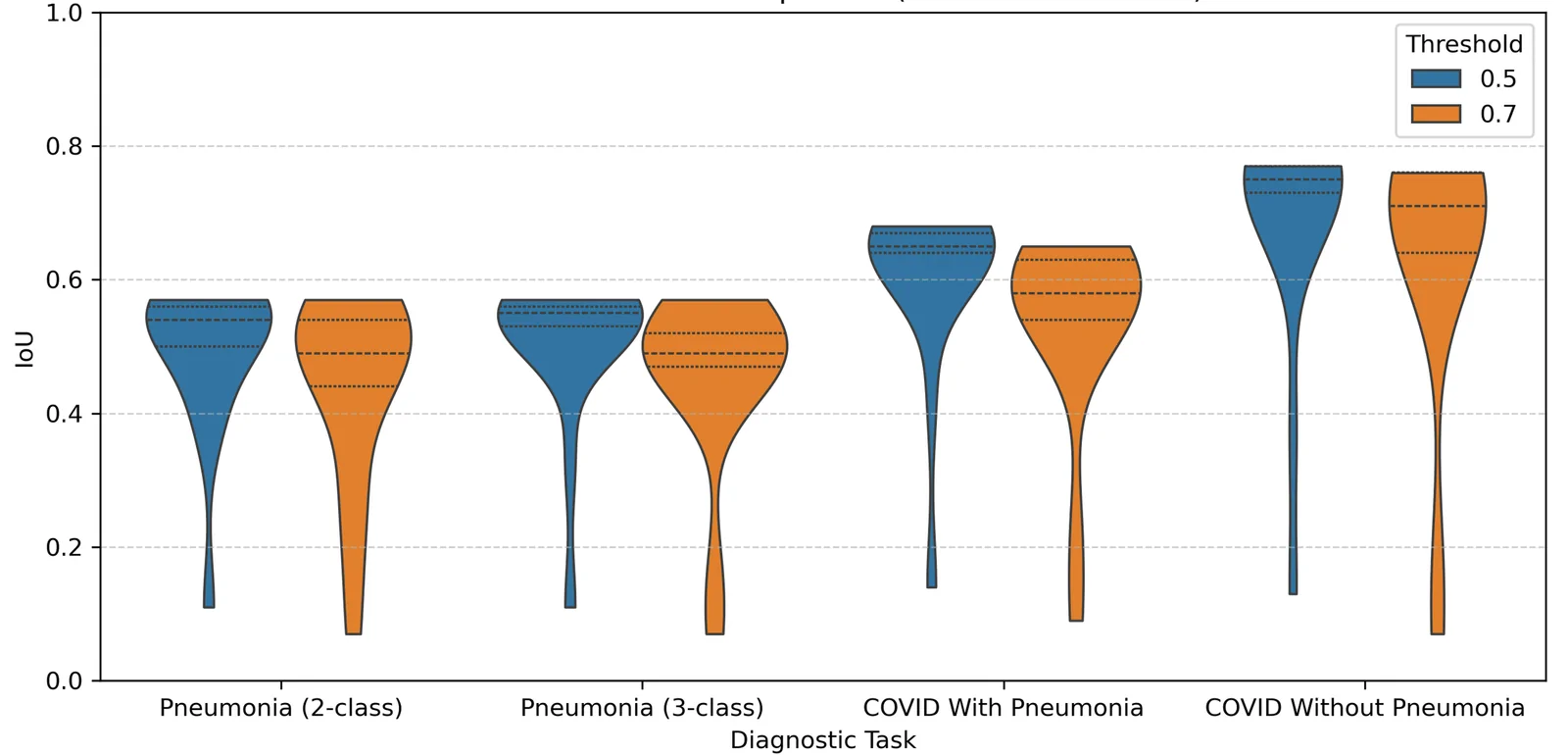
Violin plots showing the distributions of IoU scores across diagnostic tasks at relevance thresholds t = 0.5 and t = 0.7. IoU: intersection over union; COVID-19: coronavirus disease 2019; t: relevance threshold used to binarize the explanation map before calculating IoU

While Table 3 reports mean IoU values summarized by explainability rule, diagnostic task, and relevance threshold, summary statistics alone can obscure important distributional effects. To better characterize how relevance thresholding influences spatial alignment between explainability heatmaps and lung anatomy, the full distribution of IoU values was further analyzed using violin plots, as shown in Figure 8.

Figure 8 presents the distribution of IoU scores across all explainability rules for each diagnostic task, separated by relevance thresholds t = 0.5 and t = 0.7. Unlike the tabulated means, this visualization reveals not only central tendencies but also variance, skewness, and the presence of low- and high-IoU tails, enabling a more nuanced comparison of threshold behavior.

Across the diagnostic tasks, the lower threshold of t = 0.5 generally produced broader relevance regions and greater spatial overlap with the lung masks. In the two-class pneumonia task, IoU values spanned a wide range, with several instances falling below 0.3, demonstrating substantial variability among explainability methods. This broader distribution indicates that lung-field overlap varied substantially across attribution rules.

In contrast, higher thresholding (t = 0.7) generally reduced IoU values across diagnostic tasks while retaining only the most salient relevance regions. This pattern is consistent with the mean values reported in Table 3 and reflects the smaller spatial extent of thresholded relevance maps at the stricter threshold. Although several high-performing methods maintained comparatively strong overlap at t = 0.7, the overall distributions did not demonstrate a systematic increase in IoU relative to t = 0.5.

The paired comparison between thresholds therefore highlights the sensitivity of IoU to relevance threshold selection. Lower thresholds preserve a broader relevance footprint and generally produce greater overlap with the lung masks, whereas higher thresholds provide a stricter assessment focused on the most strongly attributed regions.

Notably, COVID-19-related diagnostic tasks demonstrated higher overall IoU values than the pneumonia-only tasks at both evaluated thresholds. CWOP exhibited the highest overall mean lung-field overlap, followed by CWP, whereas the binary and three-class pneumonia tasks showed lower mean overlap. These findings suggest that explanations associated with COVID-19 predictions were more strongly aligned with lung anatomy in the evaluated cases.

Together, these distributional findings complement the mean IoU results reported in Table 3 by demonstrating that threshold choice affects both the magnitude and distribution of lung-field overlap across methods. The results support evaluating explainability methods across multiple relevance thresholds rather than relying on a single operating point.

## Discussion

This study evaluated CNN predictions for chest X-ray classification using complementary qualitative and quantitative explainability analyses. Qualitative case analysis examined whether attribution patterns corresponded with radiographically described abnormalities in representative clinically characterized images, whereas quantitative IoU analysis measured spatial alignment between thresholded relevance maps and anatomical lung-field ROIs. These approaches address related but distinct questions: the qualitative analysis provides case-level evidence of pathology-relevant spatial correspondence, while IoU quantifies whether model attribution remains anatomically lung-centered rather than predominantly off-lung. Neither measure alone establishes independent clinical correctness, but together they provide complementary evidence regarding the plausibility and anatomical grounding of model explanations.

Comparison between binary and multi-class classification tasks

Descriptive comparison suggested differences in explainability behavior between the binary and three-class classification settings. Although both pneumonia classifiers achieved strong predictive performance, their relevance distributions showed different spatial patterns.

In the binary pneumonia task, LRP heatmaps generally produced broader relevance patterns across the lung fields. This may reflect reliance on more distributed pulmonary features when distinguishing pneumonia from normal cases. In the three-class setting, relevance patterns were somewhat more spatially constrained for the representative pneumonia analysis, while the mean lung-field IoU was only slightly higher than in the binary task.

Because no inferential statistical comparison was performed, these small numerical differences should be interpreted descriptively rather than as evidence that multi-class classification produces inherently more anatomically focused explanations. The results instead suggest that attribution behavior may vary with diagnostic task structure, a pattern that warrants confirmation in larger and independently evaluated datasets.

COVID-19 with vs. without pneumonia cases: distinct explainability signatures

The most pronounced differences in explainability behavior were observed when comparing CWP and CWOP tasks. For CWP, relevance maps frequently overlapped with regions of bilateral lung involvement, resulting in relatively high IoU scores at both thresholds. This pattern aligns with known radiographic manifestations of COVID-19-related pneumonia, where pathological features such as ground-glass opacities and consolidations are distributed throughout the lung fields [5].

Conversely, the CWOP task exhibited the highest mean IoU values in the evaluated set. Qualitatively, the corresponding heatmaps showed concentrated relevance within the pulmonary boundaries with relatively little off-lung activation. These observations suggest that model attribution for these cases was strongly aligned with pulmonary anatomy.

However, higher whole-lung IoU should not be interpreted as evidence of more accurate pathology localization. Diffuse radiographic patterns may naturally produce broader attribution within the lung fields and therefore greater overlap with a whole-lung ROI than more focal abnormalities. Accordingly, differences in IoU between COVID-19 and pneumonia cases are interpreted here as differences in anatomical lung-field alignment rather than differences in lesion-localization accuracy. The qualitative case analysis provides a separate pathology-relevant comparison with reported radiographic findings.

Threshold effects and explainability reliability

Comparing relevance thresholds revealed an important trade-off between sensitivity and specificity in explanation localization. Lower thresholds (t = 0.5) preserved a larger fraction of relevance mass, resulting in broader but less precise attribution regions. In contrast, higher thresholds (t = 0.7) retained only the most salient relevance values and generally produced lower IoU scores because fewer relevance pixels remained available to overlap the lung masks. Nevertheless, several structured attribution methods maintained comparatively strong localization at the stricter threshold, particularly in the COVID-19-related tasks.

This effect was also evident in the COVID-19 classification tasks, which maintained comparatively high IoU values at both evaluated thresholds. Although increasing the threshold to t = 0.7 reduced overall spatial overlap with the lung masks, several explainability methods continued to demonstrate relatively strong localization. The violin plots further illustrate the distributional changes associated with threshold selection, including shifts in central tendency and variability across diagnostic tasks.

These findings underscore the importance of threshold selection when evaluating explainability, as measured localization can vary substantially depending on the binarization threshold. Within the range examined in this study, t = 0.5 generally produced greater spatial overlap with the lung masks, whereas t = 0.7 provided a stricter evaluation focused on the most strongly attributed regions. Evaluating multiple thresholds therefore provides a more comprehensive characterization of explanation localization than reliance on a single operating point.

Qualitative heatmap analysis and anatomical plausibility

Beyond quantitative lung-field overlap, qualitative inspection demonstrated differences in how attribution methods represented the radiographically described findings in representative cases. Several methods produced relevance patterns that visually corresponded with reported pulmonary opacities or consolidations while remaining predominantly within the thoracic region, whereas others produced diffuse, grid-like, or off-lung attribution.

The qualitative and quantitative evaluations therefore provided complementary information. Case-level comparison with radiographic findings assessed pathology-relevant spatial correspondence, whereas lung-field IoU assessed anatomical grounding and the extent of obvious off-lung attribution. Anatomical alignment alone does not establish that an attribution map has correctly localized the underlying lesion; however, attribution dominated by non-pulmonary regions can identify explanations that are less anatomically plausible and may reflect reliance on irrelevant image features [28,29].

Implications and future directions

Taken together, these findings demonstrate that explainability in medical imaging should be evaluated using both qualitative and quantitative approaches and across multiple diagnostic contexts. The observed differences between binary and multi-class classification tasks, as well as between the COVID-19 subcategories, suggest that explainability behavior is highly task-dependent.

Future work could extend this framework by incorporating expert-annotated pathology regions as an additional reference standard, comparing LRP with other attribution methods such as gradient-weighted class activation mapping (Grad-CAM) [30], and evaluating explanation consistency across longitudinal scans. Additionally, investigating explainability under distribution shift could provide further insight into the robustness and reliability of attribution methods in real-world clinical settings.

Limitations

Several limitations should be considered when interpreting these findings. First, the compact CNN architecture and 64 × 64-pixel input resolution were selected for computational efficiency and consistency across experiments but may limit sensitivity to subtle radiographic findings compared with higher-resolution or deeper pretrained architectures. Second, both classifier datasets were partitioned at the image level. Because patient identifiers were not used to enforce patient-level separation, the possibility that correlated images from the same patient occurred across training and held-out subsets cannot be completely excluded.

Third, the lung-field ROIs used for quantitative IoU analysis were manually delineated by the study team rather than independently segmented by expert radiologists. Although case-specific Radiopaedia interpretations provided clinical context for the qualitative analysis, the quantitative masks represented anatomical lung fields rather than expert-annotated pathological lesions. Consequently, IoU measures anatomical lung-field alignment and should not be interpreted as lesion-level pathology-localization accuracy. This distinction is particularly relevant when comparing diffuse radiographic patterns with more focal abnormalities, because broader pulmonary attribution may inherently overlap a greater proportion of a whole-lung ROI.

Fourth, the supplementary explainability set was small and included only 13 clinically characterized chest X-rays, limiting generalizability of the qualitative comparisons. The classifier datasets were publicly available datasets and may not capture the variability in imaging acquisition, patient demographics, disease prevalence, and clinical environments encountered in real-world practice. Finally, comparisons of IoU across attribution methods and diagnostic settings were descriptive, and no inferential hypothesis testing was performed; therefore, small differences between methods or tasks should not be interpreted as statistically significant. Future studies should incorporate independent patient-level datasets, expert-annotated pathology masks, radiologist assessment of attribution maps, higher-resolution imaging, and additional XAI techniques.

## Conclusions

This study presents a framework for evaluating explainability in chest X-ray classification by combining predictive performance assessment with complementary qualitative and quantitative attribution analyses. Qualitative comparison of representative clinically characterized cases demonstrated that several attribution methods produced relevance patterns corresponding with reported radiographic abnormalities, while quantitative IoU analysis characterized the extent to which attribution remained spatially aligned with anatomical lung fields rather than obvious off-lung regions. Across the evaluated models and images, attribution behavior varied according to XAI method, diagnostic setting, and relevance threshold.

These two forms of evaluation should be interpreted distinctly. Qualitative case analysis provides pathology-relevant evidence of spatial correspondence in representative images, whereas whole-lung IoU measures anatomical alignment and does not establish lesion-specific localization accuracy. Within these limitations, combining pathology-informed qualitative analysis with quantitative anatomical evaluation provides a more complete assessment of explanation plausibility than either approach alone. Future validation using expert-annotated pathology regions, independent radiologist assessment, and external patient-level datasets will be important for determining the clinical reliability of these explanation methods. With such validation, explainable chest X-ray classification systems could ultimately support more transparent clinical decision-making, particularly in rural and under-resourced settings where access to specialized radiologic expertise may be limited.
